# Supplementary material for: Stars2Cells: Astrometric Tracking of Neurons Across Imaging Sessions
Source: bioRxiv. 2026 Jul 8:2026.07.03.736144. Preprint. [Version 1] doi: 10.64898/2026.07.03.736144 (PMC13370372; doi:10.64898/2026.07.03.736144)
Supplement: Supplement 1 [file NIHPP2026.07.03.736144v1-supplement-1.pdf]

bioRxiv preprint doi: https://doi.org/10.64898/2026.07.03.736144; this version posted July 8, 2026. The copyright holder for this preprint (which was not certified by peer review) is the author/funder, who has granted bioRxiv a license to display the preprint in perpetuity. It is made available under a CC-BY 4.0 International license.

Supplementary Information

Supplementary Note 1: Ownership Guard Uniqueness

Proposition 1 (Unique Emission). *Let $Q = \{P_a, P_b, P_c, P_d\}$ be a quad with six pairwise distances. Under the ownership guard, Q is emitted exactly once across the entire diagonal set: from the single diagonal equal to its longest pairwise edge.*

Proof. Define the ownership diagonal of Q as the pair (i^*, j^*) achieving the maximum pairwise distance:

$$(i^*, j^*) = \arg \max_{(i,j) \in \binom{Q}{2}} |P_i - P_j| \tag{S1}$$

When processing diagonal (d_1, d_2), the ownership guard emits Q only if:

$$\max_{(i,j) \in \binom{Q}{2}} |P_i - P_j| \leq |P_{d_2} - P_{d_1}| \cdot (1 + \epsilon), \quad \epsilon = 10^{-6} \tag{S2}$$

This condition is satisfied if and only if (d_1, d_2) *is* the longest edge (within floating-point tolerance). For generic point configurations (no two pairwise distances exactly equal), exactly one of the six edges is longest, so Q is emitted exactly once. Exact ties occur with probability zero for real-valued centroid coordinates. □

Complexity reduction. Without the ownership guard, each quad could be emitted from up to 6 diagonals (one per pairwise edge that appears in the sampled diagonal set), requiring an $O(Q \log Q)$ global deduplication pass via np.unique. With the guard, the output is duplicate-free by construction and the deduplication pass is eliminated entirely.

Supplementary Note 2: $\sqrt{N}$ Threshold Scaling

The optimal descriptor similarity threshold τ must balance two forces: too low and same-cell matches are rejected (low recall); too high and false matches dominate (low precision). The per-animal calibration in Step 1.5 finds the optimum τ at each N by sweeping thresholds on sampled quad subsets, and the empirical scaling across the four neuron-count tiers tested is well-fit by $\tau = C\sqrt{N}$. Below we derive this scaling from

bioRxiv preprint doi: https://doi.org/10.64898/2026.07.03.736144; this version posted July 8, 2026. The copyright holder for this preprint (which was not certified by peer review) is the author/funder, who has granted bioRxiv a license to display the preprint in perpetuity. It is made available under a CC-BY 4.0 International license.

the mean-KNN-distance behavior of the local-diagonal class, and show why the alternative scaling implied by 4-D descriptor density is non-binding in the operating scenario.

Setup. S2C builds quads from two diagonal classes: (i) KNN-local diagonals connecting each neuron to its k nearest neighbors, and (ii) random long-range diagonals spanning the FOV. The two classes have very different diagonal-length scaling with N and, consequently, very different descriptor-blur behavior. The threshold is set by whichever class is worse-blurred, which turns out to be the local class.

Step 1: local diagonal length scales as $N^{-1/2}$. For N points distributed in a fixed FOV of area A, the mean distance to the k-th nearest neighbor scales as

$$L_q^{\text{local}} \sim \sqrt{kA/N} \propto N^{-1/2} \qquad (\text{fixed } k, A). \tag{S3}$$

This holds for both Poisson and Poisson-disk-sampled point patterns; only the prefactor differs (Poisson-disk is more concentrated around its mean than Poisson, but both inherit the $N^{-1/2}$ exponent from constant-density scaling). The intuition is direct: in a denser field, your nearest neighbor is simply closer.

Step 2: same-cell descriptor blur scales as $\sqrt{N}$. Per-neuron centroid jitter of standard deviation σ_j propagates to descriptor space as $\sigma_d \approx \sqrt{2}\,\sigma_j / L_q$ (Methods). Substituting Step 1:

$$\sigma_d^{\text{local}} \approx \frac{\sqrt{2}\,\sigma_j}{\sqrt{kA/N}} = \sigma_j \sqrt{\frac{2N}{kA}} \propto \sqrt{N}. \tag{S4}$$

The intuition: at high density the same absolute jitter occupies a larger fraction of the (now shorter) diagonal length, and that ratio is exactly what propagates into descriptor space.

Step 3: the threshold tracks same-cell blur. A same-cell quad pair has descriptor distance $\sim \sigma_d$ in expectation. For same-cell matches to survive descriptor filtering, τ must exceed σ_d by a constant capture margin $\beta > 1$:

$$\tau \gtrsim \beta\,\sigma_d^{\text{local}} = \underbrace{\beta\,\sigma_j \sqrt{\tfrac{2}{kA}}}_{C} \sqrt{N}. \tag{S5}$$

The constant C absorbs the capture margin β (set by the desired same-cell capture fraction; e.g., $\beta \approx 2$ captures ∼95% of a Gaussian), the per-animal jitter level σ_j, the usable FOV area A, the KNN parameter k, and an $O(1)$ geometric prefactor relating mean KNN distance to typical local-quad diagonal length. None of these depend on N, so the leading-order behavior is exactly $\tau \propto \sqrt{N}$. The empirical per-animal calibration

bioRxiv preprint doi: https://doi.org/10.64898/2026.07.03.736144; this version posted July 8, 2026. The copyright holder for this preprint (which was not certified by peer review) is the author/funder, who has granted bioRxiv a license to display the preprint in perpetuity. It is made available under a CC-BY 4.0 International license.

of C recovers exactly this group of experimental constants.

The 4-D descriptor-density floor is non-binding An alternative starting point is descriptor-space density: $Q \propto N$ quads occupy a compact 4-D region, so the expected nearest-neighbor distance among descriptors scales as $\mathrm{NN}_d \propto N^{-1/4}$ and the false-match floor in cosine distance shrinks accordingly. This argument predicts τ should also *shrink* with N – the opposite direction from both the empirical fit and Step 3 above. The reconciliation is that same-cell blur grows with N much faster than the false-match floor shrinks; their ratio is

$$\frac{\sigma_d^{\mathrm{local}}}{\mathrm{NN}_d} \propto \frac{N^{1/2}}{N^{-1/4}} = N^{3/4}. \tag{S6}$$

Throughout the operating range, same-cell blur is the binding constraint by a margin that widens with N; the false-match floor is non-binding. False matches that survive the descriptor stage are filtered downstream by the consistency check and RANSAC, which are explicitly designed to absorb high false-match fractions.

Why long-range diagonals are insensitive to N. Random long-range diagonals span the FOV regardless of density, $L_q^{\mathrm{long}} \sim \sqrt{A}$, so their descriptor blur $\sigma_d^{\mathrm{long}} \approx \sqrt{2}\,\sigma_j/\sqrt{A}$ is independent of N. This is the design reason both classes exist. Local diagonals stabilize matching under small inter-session shifts but set the threshold; long-range diagonals carry the matching signal at high density and operate well below τ. The two-class construction is what makes per-animal calibration tractable: C only needs to track local-diagonal blur, while the bulk of useful matches come from the long-range class.

Supplementary Note 3: Coverage Remediation

The ownership guard (Supplementary Note 1) eliminates duplicate quads but can cause neurons in dense local regions to have zero or very few quads. This occurs when a neuron only participates in short diagonals, all of which have their quads claimed by longer neighboring diagonals.

Detection. For each neuron i, define coverage κ_i as the number of quads containing i. The field median coverage is $\tilde{\kappa} = \mathrm{median}(\{\kappa_i\}_{i=1}^{N})$. Neuron i is *undercovered* if:

$$\kappa_i < f_{\min} \cdot \tilde{\kappa} \tag{S7}$$

where $f_{\min} = 0.4$ by default.

Remediation. For each undercovered neuron i, all diagonals (i, j) and (j, i) in the diagonal bucket

bioRxiv preprint doi: https://doi.org/10.64898/2026.07.03.736144; this version posted July 8, 2026. The copyright holder for this preprint (which was not certified by peer review) is the author/funder, who has granted bioRxiv a license to display the preprint in perpetuity. It is made available under a CC-BY 4.0 International license.

dictionary are re-processed by _process_diagonal with enforce_ownership=False. The resulting quads are subject to all other quality filters (area ≥ 1.0, min pairwise distance, non-degeneracy) and deduplicated against the existing quad set via a Python set of sorted index tuples. Quality pruning (quad_keep_fraction) is applied to the remediation batch independently.

Guarantees. Remediation quads may duplicate quads that would have been emitted from a different diagonal under normal ownership. However, because they are deduplicated against the existing set, no quad appears twice in the final output. The trade-off is that these remediation quads lose the automatic "emitted exactly once" guarantee the ownership guard gives the main pass: because they are produced by diagonals that are not their rightful owner, their uniqueness is enforced by the explicit duplicate check rather than guaranteed by construction.

Supplementary Note 4: Computational Complexity

Step	Operation	Complexity
1 (Diag. buckets)	KD-tree + edge list	$O(N \log N + N \cdot k)$
1 (Heights)	Per-diagonal height computation	$O(\lvert\mathcal{E}\rvert \cdot N)$
1 (Quads)	Pair third points + ownership	$O(\lvert\mathcal{E}\rvert \cdot K^2)$
1.5 (Calibration)	Threshold sweep on subsets	$O(n_{\text{pairs}} \cdot n_\tau \cdot Q_{\text{sample}})$
2 (Matching)	Cosine NN search	$O(Q_r \cdot Q_t)$ or $O(Q_r \log Q_t)$ with FAISS
2.5 (RANSAC)	Batched SVD + chunked residuals	$O(K_{\text{iter}} \cdot m^2 + K_{\text{iter}} \cdot M/C)$
3 (Hungarian)	Vote matrix + assignment	$O(M \cdot 16 + n^3)$
3 (Consolidation)	Transitive chaining	$O(S \cdot n)$

where N = neurons, $k = k_{\text{local}} + k_{\text{random}}$, $\lvert\mathcal{E}\rvert$ = number of diagonals $\approx Nk/2$, K = top-K cap per diagonal, Q = total quads, M = matched quads, $n = \min(N_r, N_t)$, S = number of sessions.

Supplementary Note 5: Synthetic Degradation Model

Base field generation. For each synthetic animal, a base centroid field $\{(x_i^{(0)}, y_i^{(0)})\}_{i=1}^{N}$ is placed in a 600×600-pixel FOV by Bridson Poisson-disk sampling, producing a blue-noise (well-separated, non-clustered) layout. The minimum spacing $r_{\min}$ defaults to 55% of the theoretical hex-packing maximum for

bioRxiv preprint doi: https://doi.org/10.64898/2026.07.03.736144; this version posted July 8, 2026. The copyright holder for this preprint (which was not certified by peer review) is the author/funder, who has granted bioRxiv a license to display the preprint in perpetuity. It is made available under a CC-BY 4.0 International license.

the requested N within the usable FOV (margin = 10 px), so density automatically scales with neuron count. A fallback rejection-sampling step with relaxed $r_{\min}$ (factor 0.8) handles edge cases where Bridson cannot place all requested points in the first pass.

Per-neuron base jitter. Independent of session-to-session perturbations, each neuron carries a stable per-neuron centroid offset $(\delta x_i, \delta y_i) \sim \mathcal{N}(0, \sigma_b^2)$ with $\sigma_b = 0.5$ px, modeling systematic CNMF centroid bias that persists across sessions for that neuron. This is added in addition to a per-session perturbation $\sigma_p = 0.2$ px drawn fresh each session.

Session generation. For session $s \in \{1, \ldots, S\}$ (where $S = 5$), the base field is transformed in the following fixed order:

1. **Rotation:** $\begin{pmatrix} x_i' \\ y_i' \end{pmatrix} = R(\theta_s) \begin{pmatrix} x_i^{(0)} - \bar{x} \\ y_i^{(0)} - \bar{y} \end{pmatrix} + \begin{pmatrix} \bar{x} \\ \bar{y} \end{pmatrix}$, where $R(\theta) = \begin{pmatrix} \cos\theta & -\sin\theta \\ \sin\theta & \cos\theta \end{pmatrix}$, with θ_s accumulating across sessions in Tiers A and B and following the random-walk schedule in Tier C.

2. **Translation:** $(x_i', y_i') \leftarrow (x_i' + t_x^{(s)},\ y_i' + t_y^{(s)})$, similarly accumulating per tier.

3. **Clip-to-FOV:** any neuron whose transformed position falls outside $[0, 600]^2$ is removed, modeling loss off the sensor edge after large transforms (this is what makes large rotation/translation conditions genuinely hard rather than merely shifted).

4. **Dropout (sessions ≥ 2 only):** a fraction δ_s of remaining neurons is randomly removed, modeling CNMF detection failure. *No new neurons are spawned* – this matches the biological fact that ROIs can fail to be detected in a given session, but new cells do not spontaneously appear.

5. **Jitter:** $(x_i', y_i') \leftarrow (x_i' + \delta x_i + \epsilon_x^{(s)},\ y_i' + \delta y_i + \epsilon_y^{(s)})$, where $(\delta x_i, \delta y_i)$ is the stable per-neuron base offset and $\epsilon_x^{(s)}, \epsilon_y^{(s)} \sim \mathcal{N}(0, \sigma_p^2)$ is the per-session perturbation, applied only to neurons that survived clipping and dropout.

6. **Ground-truth capture:** the surviving base indices (before permutation) are stored as ground_truth _base_ids in the per-file ground-truth dictionary.

7. **ID permutation:** ROI identifiers are randomly shuffled to prevent matching by index.

bioRxiv preprint doi: https://doi.org/10.64898/2026.07.03.736144; this version posted July 8, 2026. The copyright holder for this preprint (which was not certified by peer review) is the author/funder, who has granted bioRxiv a license to display the preprint in perpetuity. It is made available under a CC-BY 4.0 International license.

Supplementary Note 6: CellReg Reimplementation and Validation

ROI-based matching via a probabilistic model. CellReg [9] models the probability that neurons i (session 1) and j (session 2) are the same cell as:

$$P(\text{same} \mid \rho_{ij}, d_{ij}) = \frac{P(\rho_{ij}, d_{ij} \mid \text{same}) \cdot P(\text{same})}{P(\rho_{ij}, d_{ij})} \tag{S8}$$

where ρ_{ij} is the spatial footprint correlation and d_{ij} is the centroid distance. The likelihoods are modeled as:

$$P(\rho, d \mid \text{same}) = f_{\text{same}}(\rho) \cdot g_{\text{same}}(d) \tag{S9}$$

$$P(\rho, d \mid \text{diff}) = f_{\text{diff}}(\rho) \cdot g_{\text{diff}}(d) \tag{S10}$$

where $g_{\text{same}}(d)$ is modeled as a Rayleigh distribution (centroid distances for matched neurons) and $g_{\text{diff}}(d)$ as approximately uniform over the FOV. The correlation distributions $f_{\text{same}}(\rho)$ and $f_{\text{diff}}(\rho)$ are estimated non-parametrically from the data. Parameters are fit via expectation-maximization over the observed pairs.

Python reimplementation. The step-by-step port of CellReg's spatial-correlation pathway, its cluster_cells final-assignment step (which differs from a one-shot Hungarian solve), and its pair-for-pair validation against native MATLAB CellReg are given in Methods ("ROI-based matching reimplementation"). The probabilistic model above is what those steps implement.

Parallelization. The validated reimplementation was wrapped in an embarrassingly-parallel batch driver (Python's concurrent.futures.ProcessPoolExecutor, up to 50 workers, each pinned to a single BLAS thread) so the full benchmark runs on one workstation; the algorithm is unchanged and only its execution is parallelized.

Supplementary Note 7: Full Per-Condition Benchmark Statistics

Table 1 expands the pooled and per-neuron-count summaries in the main text (Fig. 4g) to all 32 (condition × neuron-count) benchmark cells. S2C's F1 advantage over ROI-based matching is positive and significant in every cell, with the smallest gaps on the mildest perturbation (Tier-A translation) and the largest on the high-displacement Tier-B/C conditions.

bioRxiv preprint doi: https://doi.org/10.64898/2026.07.03.736144; this version posted July 8, 2026. The copyright holder for this preprint (which was not certified by peer review) is the author/funder, who has granted bioRxiv a license to display the preprint in perpetuity. It is made available under a CC-BY 4.0 International license.

Table 1: Per-condition S2C vs. ROI-based matching (worst-case footprint scenario). Mean F1 per tool, the paired S2C−ROI F1 difference with 95% bootstrap CI, paired Cohen's d_z, and Wilcoxon p (Holm-corrected across conditions within each neuron-count tier). $n = 40$ paired runs per cell except where CellReg returned no defined F1 (excluded per Methods); S2C precision was $\geq 97.7\%$ throughout; best-case footprint values are near-identical.

Condition	n	S2C F1 (%)	ROI F1 (%)	ΔF1 (pp) [95% CI]	d_z	p_{Holm}
$N = 100$ *neurons*						
A: rotation	40	99.6	76.0	+23.5 [+15.2, +32.4]	+0.82	2.3×10^{-9}
A: translation	40	99.4	97.8	+1.6 [+0.8, +2.6]	+0.57	9.4×10^{-4}
A: combined	40	99.4	68.8	+30.6 [+22.0, +39.3]	+1.10	1.5×10^{-11}
B: dropout	40	99.9	47.9	+52.0 [+46.0, +57.6]	+2.68	1.5×10^{-11}
B: drift	35	99.8	19.3	+80.6 [+74.9, +85.9]	+4.84	1.7×10^{-10}
B: rotation	39	99.9	14.4	+85.5 [+79.9, +90.1]	+5.15	1.8×10^{-11}
B: combined	39	99.8	12.3	+87.5 [+83.2, +91.2]	+6.75	1.8×10^{-11}
C: random walk	40	99.7	23.1	+76.6 [+70.9, +82.0]	+4.17	1.5×10^{-11}
$N = 250$ *neurons*						
A: rotation	40	99.9	69.0	+30.9 [+21.5, +41.0]	+0.98	1.5×10^{-11}
A: translation	40	99.9	97.4	+2.5 [+1.2, +4.0]	+0.54	6.3×10^{-7}
A: combined	40	99.9	62.0	+37.9 [+28.7, +47.0]	+1.24	1.5×10^{-11}
B: dropout	40	99.9	36.8	+63.1 [+59.1, +67.1]	+4.81	1.5×10^{-11}
B: drift	38	99.9	15.4	+84.5 [+78.7, +89.9]	+4.86	1.6×10^{-7}
B: rotation	40	99.9	7.6	+92.4 [+89.9, +94.5]	+12.27	1.5×10^{-11}
B: combined	39	99.9	6.8	+93.1 [+91.2, +94.8]	+15.58	1.6×10^{-7}
C: random walk	40	99.9	20.1	+79.8 [+76.5, +82.8]	+7.63	1.5×10^{-11}
$N = 500$ *neurons*						
A: rotation	40	97.2	54.7	+42.4 [+33.4, +51.3]	+1.45	1.6×10^{-11}
A: translation	40	97.4	92.9	+4.5 [+2.3, +6.9]	+0.61	4.6×10^{-3}
A: combined	40	97.2	45.0	+52.2 [+44.2, +59.7]	+2.07	1.5×10^{-11}
B: dropout	40	97.0	30.5	+66.5 [+63.9, +69.0]	+7.86	1.5×10^{-11}
B: drift	35	97.0	11.3	+85.6 [+81.5, +89.5]	+6.97	1.2×10^{-10}
B: rotation	40	96.7	6.3	+90.4 [+88.1, +92.4]	+12.78	1.5×10^{-11}
B: combined	39	96.8	5.2	+91.6 [+90.2, +92.9]	+21.03	1.5×10^{-11}
C: random walk	40	96.7	15.4	+81.3 [+78.5, +83.9]	+9.19	1.5×10^{-11}
$N = 1{,}000$ *neurons*						
A: rotation	40	94.3	39.8	+54.4 [+45.9, +62.7]	+1.97	1.5×10^{-11}
A: translation	40	94.9	84.3	+10.6 [+6.8, +14.6]	+0.82	2.6×10^{-4}
A: combined	40	94.2	32.7	+61.5 [+55.0, +67.4]	+3.07	1.5×10^{-11}
B: dropout	40	99.3	23.2	+76.1 [+75.0, +77.0]	+22.98	1.5×10^{-11}
B: drift	38	100.0	7.9	+92.1 [+89.2, +94.8]	+10.12	2.2×10^{-11}
B: rotation	40	99.8	4.4	+95.3 [+94.0, +96.6]	+22.20	1.5×10^{-11}
B: combined	40	97.4	3.4	+93.9 [+92.0, +95.6]	+15.70	7.1×10^{-8}
C: random walk	40	98.4	10.5	+87.9 [+85.9, +89.8]	+13.90	1.5×10^{-11}
